# Suprasellar region: view during an endoscopy fenestration of a suprasellar arachnoid cyst

**DOI:** 10.11604/pamj.2020.35.25.19691

**Published:** 2020-02-03

**Authors:** Sidi Salem-Memou, Nejat Boukhrissi

**Affiliations:** 1 Department of Neurosurgey, National Hospital Centre of Nouakchott, Nouakchott, Mauritania

**Keywords:** Arachnoid cysts, suprasellar cysts, neuroendoscopy

## Image in medicine

Arachnoid cysts are intra-arachnoid fluid formations containing cerebrospinal fluid. About 66% of arachnoid cysts are located in the temporal fossa. The suprasellar location is relatively rare, representing 5%-12% of intracranial arachnoid cysts in the general population and 16% in the pediatric population (1-8). They are most often congenital and progressively growing due to an abnormality of the Liliequist membrane or the interpeduncular cistern. The management of suprasellar arachnoid cysts requires a perfect understanding of their anatomy and physiopathogeny. Endoscopic ventriculo-cysto-cisternostomy is an interesting alternative in the management of this pathology. A 7-year-old girl with no significant pathological history, who had visual problems associated with headache for 13 months. At admission, the examination noted a patient conscious with a very ataxic walking without sensitivomotor deficit. Visual acuity was 3/10 on both sides. Brain magnetic resonance imaging (A,B) showed an important suprasellar expansive process, well limited, in hyposignal in sequences T1 (A) and hypersignal in sequence T2 (B) identical to CSF signal, without enhancement after gadolinium injection . This process pushes up the optic chiasma and blocks Monro’s foramens, achieving obstructive hydrocephalus. The patient was operated by transventricular approach with endoscopic ventriculocystocisternostomy (VCC) (C). During the endoscopic procedure, the anatomical view of the skull base was demonstrative. From the interior of the cyst (C), we have identified the following anatomical structures: optic nerve, pituitary stalk, internal carotid arteries, pituitary gland, posterior communicating arteries, posterior cerebral arteries, basilar artery, dorsum sellae. The evolution was marked by a good clinical improvement with a regression of signs of intracranial hypertension and improvement of walking during the first postoperative week. Visual recovery wasn’t observed until the 4th week.

**Figure 1:**
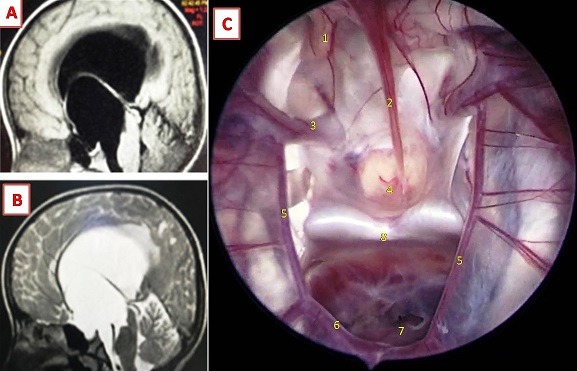
A) Sagital T1; B) T2-weighted MR image showing a large suprasellar arachnoid cyst with marked obstructive hydrocephalic changes; C) view of the suprasellar region during the endoscopic ventriculocystocisternostomy, showing the right optic nerve (1), pituitary stalk (2), internal carotid arteries (3), pituitary gland (4), posterior communicating arteries (5), posterior cerebral arteries (6), basilar artery (7), dorsum sellae (8)

